# Multi-Scale Structural Regulation of Boron-Doped Diamond via Doping, Modification, and Annealing for Water Pollutant Sensing

**DOI:** 10.3390/nano16130834

**Published:** 2026-07-07

**Authors:** Xue Wang, Shuxian Leng, Xiang Yu, Shengmao Lu, Junsheng Wang

**Affiliations:** School of Materials Science and Technology, China University of Geosciences (Beijing), Beijing 100083, China; 13522938212@163.com (X.W.); 1003230201@email.cugb.edu.cn (S.L.); 15647958487@163.com (S.L.); 19898858838@163.com (J.W.)

**Keywords:** nanostructured thin films, boron-doped diamond electrode, structural regulation, water pollutant detection, nanomaterial modification, post-annealing treatment, electrochemical sensing

## Abstract

This review covers literature published up to June 2026. Detecting various water pollutants quickly and reliably remains a challenge. Boron-doped diamond (BDD) electrodes, particularly when fabricated as nanostructured thin films such as nanocones or nanowalls, offer a wide electrochemical window, low background current, and excellent chemical stability, making them promising tools for electrochemical sensing. However, unmodified BDD electrodes face an inherent trade-off among conductivity, active site density, and interfacial stability, a phenomenon termed herein the “sensitivity-selectivity-stability triangle bottleneck”, which severely limits practical performance. In this review, we demonstrate how multi-scale structural regulation can circumvent this bottleneck. Specifically, a triple strategy comprising boron doping, surface modification, and post-annealing treatment is proposed and evaluated. First, the effect of boron doping level on conductivity and active site density is discussed. Second, two common surface modification approaches are examined: carbon nanomaterials (which increase surface area and form conductive networks) and metal nanoparticles (which enhance catalytic activity and interfacial charge transfer). Third, post-annealing is highlighted as a key synergistic step that locks the modified layer and stabilizes the interface. Together, these three components form an integrated framework. To provide concrete guidance, the performance of each strategy is compared for representative water pollutants, including heavy metal ions, phenolic compounds, and emerging contaminants such as antibiotics and pesticides, with emphasis on sensitivity, selectivity, and stability. Representative detection limits achieved include 0.01 μg/L for Pb^2+^, 5 nM for acetaminophen, and 0.32 fM for PCB-77, demonstrating the effectiveness of the triple structural regulation strategy. Finally, in line with the theme of this Nanomaterials Special Issue on nanostructured thin films, current challenges in structural regulation are summarized, and future directions, including multi-parameter optimization, AI-assisted high-throughput screening, and real-world testing, are outlined. The goal is to offer practical structure-performance guidelines for designing BDD-based electrochemical sensors that are both high-performing and durable.

## 1. Introduction

Water pollution represents a critical global environmental challenge. A wide range of contaminants such as heavy metals, phenolic compounds, pesticides, and antibiotics are discharged into aquatic environments, posing significant risks to both ecosystems and human health [1,2]. This review systematically surveys the literature from the past two decades, with emphasis on advances reported up to June 2026. Conventional detection methods, such as gas chromatography, liquid chromatography, and mass spectrometry, while highly accurate, require expensive instrumentation, complex sample preparation, and specialist training, limiting their suitability for on-site and real-time monitoring. Therefore, fast, reliable, and portable electrochemical sensing technologies are urgently needed for water quality monitoring on site [3,4].

Electrochemical sensors are suitable candidates for this job due to their low cost, rapid response, and potential for miniaturization and portability [3,4]. Among the various electrode materials, boron-doped diamond (BDD) has emerged as a particularly advantageous platform. It offers a very wide electrochemical window, very low background current, and exhibits excellent resistance to fouling and corrosion [2,5,6,7,8]. Nanostructuring BDD into thin-film architectures, including nanocones, nanowalls, and nanorods, substantially enlarges the electrochemically active surface area and facilitates mass transport, thereby further enhancing sensing performance [9,10,11]. The benefits of nanostructuring are threefold: (i) the high specific surface area of nanostructured BDD (typically 10–100 times that of planar BDD) provides abundant active sites for analyte adsorption and electrochemical reactions; (ii) the nanoscale dimensions facilitate efficient charge transport by reducing diffusion distances and enhancing electron transfer kinetics; and (iii) the hierarchical surface morphology promotes analyte enrichment through capillary effects and enhanced local mass transfer [9,10,11]. These intrinsic nanostructures, together with externally introduced carbon/metal nanomaterials (discussed in Section 3.2 and Section 3.3), constitute the multi-scale structural regulation framework of this review. The unique properties of BDD electrodes have enabled their successful application in detecting a wide range of environmental contaminants, including pesticides, heavy metals, pharmaceuticals, and industrial pollutants [3,4,9,10,12,13].

However, an inherent trade-off exists among three critical performance metrics: electrical conductivity, the density of active sites, and interfacial stability. Three factors must be balanced in the design of BDD electrodes. First, increasing the boron doping concentration enhances conductivity and carrier density, but may narrow the potential window and reduce the relative density of active sites due to the formation of non-diamond *sp*^2^ carbon phases [6,7,8,14,15,16,17]. Second, modifying the BDD surface with carbon nanomaterials or metal nanoparticles significantly increases the electroactive surface area and introduces additional catalytic sites. However, these modification layers are prone to aggregation, uneven dispersion, or detachment during long-term operation, compromising electrode lifetime [18,19,20,21,22,23]. Third, post-annealing treatment can strengthen the adhesion of the modification layer and stabilize the interface, but annealing may also cause unwanted sintering of nanoparticles or oxidative etching of the diamond surface if the parameters are not properly optimized [11,24,25,26,27]. These interlinked challenges constitute what we term the “sensitivity-selectivity-stability triangle bottleneck”, which represents a central obstacle to the practical deployment of BDD-based sensors.

Over the past decade, three main strategies have been explored to overcome this bottleneck: tuning the boron doping level, modifying the BDD surface with functional nanomaterials, and applying post-annealing treatments. While post-annealing is widely employed as a fabrication step in BDD electrode preparation, as demonstrated by the two-step annealing process used for Au nanoparticles on BDD [28] and the annealing-enhanced Cu-BDD electrodes for nitrate reduction [25], the systematic mechanism-parameter-performance relationships of annealing, and its synergistic coupling with doping and modification, have not been critically reviewed in the context of electrochemical sensing [2,3,6,19,29,30,31]. This review addresses this gap by treating annealing not merely as a preparative step, but as an independent regulatory strategy that can be systematically optimized.

Each of these approaches has been reviewed in the literature, but largely in isolation [3,6,19,30,31]. Einaga (2022) provided a comprehensive account of the fundamental electrochemical properties of BDD, organized around three parameters, i.e., surface termination, crystal orientation, and doping level [6]. Several reviews have summarized the use of carbon nanomaterials such as carbon nanotubes, graphene, and carbon nanospheres to enhance sensitivity through increased surface area and improved electron transfer [18,19,21,32]. Metal nanoparticle decoration has also been extensively reviewed, particularly for the detection of heavy metal ions [17,20,30]. More recently, Mijajlović et al. (2025) reviewed BDD electrodes for toxin sensing in environmental samples, covering pesticides, heavy metals, pharmaceuticals, and industrial pollutants [2]. Kramplová et al. (2025) summarized voltammetric detection techniques using carbon-based electrodes, including BDD, for pesticide determination in water [33].

Other recent reviews have addressed specific aspects of BDD electrochemistry from different perspectives: Chen et al. (2026) focused on BDD for environmental remediation (electrochemical degradation) with a structure-property scheme covering doping, surface termination, morphology, and composites [34]; Hrdlička et al. (2023) comprehensively covered modification methods for BDD electrodes across diverse applications, including biosensing, electrosynthesis, and CO_2_ reduction [19]; Shellaiah and Sun (2022) restricted their scope to diamond-based electrodes for metal ions and anions [30]; Baluchová et al. (2019) summarized BDD electroanalysis of organic compounds and biomolecules for the period 2009–2018 [31]; Joshi et al. (2022) reviewed environmental applications of BDD electrochemical sensors from an application-focused perspective [3]. However, what remains missing from the literature is a systematic overview that integrates all three strategies of doping, modification, and annealing into a unified framework for water pollutant sensing [2,3,6,19,31]. In particular, none of the existing reviews have treated post-annealing as an independent regulatory strategy with systematic mechanism-parameter-performance analysis, nor have they provided pollutant-type-specific comparisons under a unified structural regulation framework [2,3,6,31].

Furthermore, most existing reviews on BDD-based sensors have predominantly focused on heavy metal ions [3,30]. However, the field has progressively expanded to encompass organic pollutants such as phenolic compounds, antibiotics, pesticides, and other emerging contaminants [2,31]. Recent advances include the development of nano-foam gold-modified BDD aptasensors for ultrasensitive detection of endocrine disruptors like 17β-estradiol [35], B:CNWs/BDD composite electrodes for herbicide detection in food and water samples (Sain, et al. [36]), and integrated sensing platforms for emerging contaminants [37,38,39]. Yet there is no comprehensive review that covers different types of water pollutants from heavy metals to phenolics to antibiotics and pesticides under the same umbrella of structural regulation.

The distinctive contributions of this review, which distinguish it from the existing reviews discussed above, are threefold: (i) we treat post-annealing as an independent regulatory strategy with systematic mechanism-parameter-performance analysis, rather than merely a preparative step mentioned in passing; (ii) we propose a “doping-modification-annealing” triple synergistic framework that integrates all three strategies into a unified optimization logic; and (iii) we provide pollutant-type-specific comparisons under the same structural regulation umbrella, a comparative approach not found in previous reviews.

To address this gap, our main scientific question is: How can multi-scale structural regulation of boron doping, nanomaterial modification (carbon and metal), and post-annealing treatment be systematically engineered to overcome the sensitivity-selectivity-stability triangle bottleneck, enabling BDD electrodes to achieve superior performance in detecting diverse water pollutants? To answer that question, we propose and evaluate a “doping-modification-annealing” triple strategy as an integrated framework.

The review is organized as follows. Section 2 briefly introduces the key performance metrics for evaluating BDD-based electrochemical sensors. Section 3 examines the three regulatory strategies in detail: boron doping (Section 3.1), carbon nanomaterials (Section 3.2), and metal nanoparticles (Section 3.3), with a comparative summary of their respective limitations (Section 3.4). Section 4 focuses on post-annealing as a synergistic tool, covering the influence of annealing parameters on the structure and properties of the modified layer (Section 4.1), its effect on interfacial stability and long-term sensing behavior (Section 4.2), and the multi-parameter coupling optimization logic (Section 4.3). Section 5 presents representative case studies organized by pollutant type of heavy metals, phenolic compounds, and emerging contaminants, with systematic performance comparison tables. Section 6 discusses the remaining challenges, trade-offs, and future directions, including AI-assisted design, real-world validation, and integrated sensing-degradation platforms. Section 7 concludes with a summary of findings and practical design guidelines for BDD-based electrochemical sensors.

## 2. Fundamentals of Sensing Performance Evaluation

Assessing the effectiveness of structural regulation strategies for BDD electrodes requires a set of well-established performance metrics: sensitivity, selectivity, stability, linear range, and limit of detection (LOD) [6,31,40]. Briefly, sensitivity reflects the current response per unit concentration change and is governed by the electroactive surface area, charge-transfer kinetics, and analyte enrichment efficiency [17,41,42]. Selectivity describes the ability to discriminate the target analyte from interferents, as indicated by peak separation potential and current retention [17,24,43]. Stability encompasses cycling reproducibility, storage longevity, and anti-fouling ability in complex media [19,32]. Linear range is the concentration interval over which the response is linearly proportional to concentration, while LOD is the lowest reliably distinguishable concentration, determined by the signal-to-noise ratio [6,40,44]. These metrics are not independent; they often trade off against each other—a central theme of the “sensitivity-selectivity-stability triangle bottleneck” introduced in Section 1.

Different water pollutants impose distinct demands on these metrics, and these differences directly inform the selection of structural regulation strategies. Heavy metal ions (e.g., Pb^2+^, Cd^2+^) require low LOD and high sensitivity, favoring moderate boron doping combined with metal nanoparticle modification and annealing [17,41,45,46]. Phenolic compounds demand a wide potential window and anti-fouling ability, which favors low-to-moderate doping and carbon nanomaterials [31,47,48]. Antibiotics and emerging contaminants call for high selectivity and long-term stability, where annealing is strongly recommended [40,46]. Mixed pollutant systems require peak separation and anti-interference capability, typically addressed by multi-scale composite regulation [17,49]. Table 1 summarizes these pollutant-specific priorities and the corresponding recommended strategies. The following sections (Section 3 and Section 4) provide detailed discussions of each strategy, and Section 5 presents case studies that illustrate their practical performance.

## 3. Structural Modification Strategies and Their Impact on Sensing Performance

### 3.1. Boron Doping Regulation: Conductivity and Potential Window via Sensitivity and Selectivity

**Figure 1:**
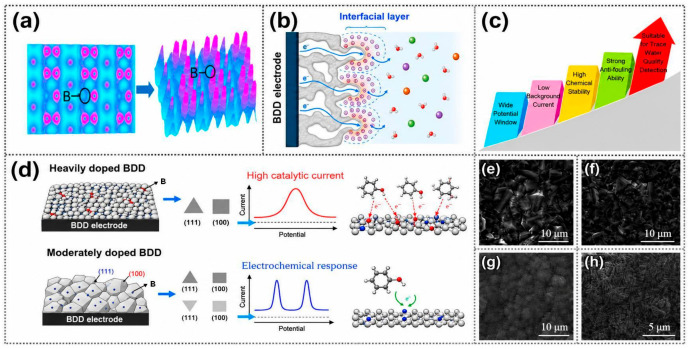
(**a**) Atomic structure of a relaxed boron-doped diamond electrode slab B denotes boron-doping sites. (Reprinted from Ref. [51]). (**b**) Enhanced interfacial charge-transfer pathway of BDD electrodes. Different colors and symbols represent electrolyte ions/target analytes/pollutant molecules; arrows indicate charge-transfer direction. (**c**) The representative advantages of boron-doped BDD electrodes in water quality analysis. (**d**) Original schematic illustration of boron-doping-mediated regulation of crystal facet exposure and sensing performance. (**e**–**h**) SEM images of BDD electrodes with different boron doping levels. (**e**) BDD_0.6_, (**f**) BDD_0.9_, (**g**) BDD_1.8_, and (**h**) magnified view of BDD_1.8_. (Reprinted from Ref. [15]).

The microstructure of the electrode is a key factor affecting the electrochemical sensing performance of BDD, and the boron doping concentration is one of the core parameters that regulate its surface morphology [6,7,14,15,50]. As shown in Figure 1a, the relaxed boron-doped diamond (111) slab reveals the local electronic perturbation induced by boron incorporation. As shown in Figure 1e–h, BDD_0.6_, BDD_0.9_, and BDD_1.8_ denote electrodes prepared using B-source flow rates of 0.6, 0.9, and 1.8 sccm, respectively. The progressive increase in B-source flow rate leads to a clear morphological evolution, from faceted diamond grains to a cauliflower-like surface in BDD_1.8_, which is associated with excessive boron incorporation and degradation of the diamond crystalline morphology [15]. The surface morphology of BDD electrodes varies significantly with boron doping concentration. At low boron concentrations, the BDD grains exhibit typical cubic or octahedral diamond structures with large grain sizes and clear grain boundaries. As the doping concentration increases, diamond grain growth is inhibited, the grain size decreases, and the surface roughness and grain boundary density increase significantly, even leading to nanoscale whiskers or dense micro-nanocrystalline structures [8,15,43]. This boron-doping-induced morphological change directly affects the electrochemically active surface area, electron transfer efficiency, and surface adsorption characteristics, thereby determining the sensitivity and selectivity of BDD electrodes in water quality sensing. Undoped diamond has a wide bandgap and low intrinsic conductivity, making it unsuitable for direct use as an efficient electrochemical sensing electrode [6,7,8]. Figure 1b demonstrates the enhanced interfacial charge-transfer pathway of BDD electrodes, while Figure 1c illustrates the key advantages of boron-doped BDD electrodes. Boron atoms have a radius similar to that of carbon and can substitute carbon atoms in the diamond lattice, forming acceptor levels and transforming diamond into a p-type semiconductor [6,7,8]. As the boron doping level increases, the hole carrier concentration in the BDD film rises, enhancing electrode conductivity and interfacial electron transfer, which in turn improves the current response during analyte redox reactions. Related studies have shown that BDD electrodes with different boron contents exhibit different carrier densities and binding energies, which further affect the oxidation peak potential and peak current intensity of the analytes [14,15,17,24,45]. Figure 1d shows that boron doping can regulate the exposed crystal facets and surface active sites of BDD electrodes. Moderate boron doping improves conductivity and electrocatalytic response while maintaining low background current, which is beneficial for selective sensing. However, excessive doping may introduce non-diamond carbon phases and reduce the potential window [24]. Therefore, boron doping should be optimized to balance conductivity, crystal facet exposure, and sensing selectivity. Thus, the boron doping level is an important structural parameter determining the sensing performance of BDD electrodes.

Moderate boron doping enhances the peak current response by increasing carrier concentration and reducing charge-transfer resistance [14,15,17,45]. Insufficient doping limits conductivity and electrocatalytic activity; excessive doping may cause lattice expansion, boron aggregation at grain boundaries, and the promotion of non-diamond carbon nucleation—processes that lead to the formation of *sp*^2^-bonded carbon phases. The mechanism of *sp*^2^ phase formation upon excessive doping can be understood as follows: as boron atoms substitute carbon in the diamond lattice at high concentrations, lattice distortion generates strain energy. This strain is preferentially relieved at grain boundaries and defect-rich regions, where carbon atoms rehybridize from *sp*^3^ to *sp*^2^ configuration, forming graphitic or amorphous carbon clusters. These *sp*^2^ phases introduce additional electronic states within the bandgap, which serve as recombination centers and facilitate non-faradaic current, thereby increasing the background current and narrowing the usable potential window [15,16,52]. The consequences include a narrowed potential window, increased background current, and reduced signal-to-noise ratio, which collectively degrade the selectivity and detection sensitivity [8,16,41].

**Figure 2:**
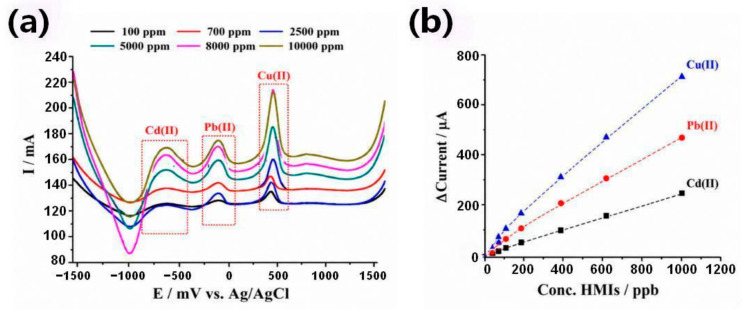
Optimization of boron doping and analytical performance of BDD electrodes for simultaneous Cd(II), Pb(II), and Cu(II) detection (Reprinted from Ref. [53]). (**a**) Comparison of stripping responses of BDD electrodes prepared with different boron doping concentrations. (**b**) Calibration plots of Cd(II), Pb(II), and Cu(II) obtained using the optimized BDD electrode.

Chen et al. [15] found that the electrochemically active surface area, oxygen evolution potential, and pollutant degradation performance of BDD electrodes do not improve monotonically with increasing boron concentration but exhibit a clear non-linear behavior. The quantitative relationship between boron doping concentration and sensing performance has been further examined in systematic studies. Chen et al. [15] further reported that the electrochemically active surface area (EASA) and oxygen evolution potential (OEP) varied non-monotonically with boron concentration. The maximum EASA of 9.366 cm^2^ (BDD_1.5_: [B]/[C] = 50,000 ppm) and OEP of 2.728 V (BDD_0.1_) were achieved at an optimized doping level, while excessive boron incorporation reduced EASA to 5.968 cm^2^ and OEP to 2.166 V, accompanied by increased *sp*^2^ phase content and lattice distortion, indicating an optimal doping window. Kim et al. [53] systematically investigated the effect of boron doping concentration on the electrochemical sensing performance of BDD electrodes for the simultaneous detection of Cd(II), Pb(II), and Cu(II). As shown in Figure 2a, the stripping responses of the three heavy metal ions increased as the boron doping concentration increased from 100 to 8000 ppm, indicating that moderate boron incorporation improved the conductivity and electrochemical activity of the BDD electrode. The highest response was obtained at 8000 ppm, whereas a further increase to 10,000 ppm resulted in a decrease in sensitivity, which was attributed to over-doping-induced lattice distortion and deterioration of crystallinity. Figure 2b further shows the calibration behavior of the optimized BDD electrode, which exhibited a good linear response over the concentration range of 1–1000 ppb for Cd(II), Pb(II), and Cu(II), confirming its promising analytical performance for simultaneous heavy-metal ion sensing. However, further increasing the doping level decreased the sensitivity, possibly due to increased *sp*^2^ phase content and reduced crystal quality.

The effect of doping on the electrochemical window is equally significant. Liu et al. [16] demonstrated that the solvent window of BDD electrodes varies with local boron doping level. Using scanning electrochemical cell microscopy (SECCM), they found that less doped facets exhibit a wider solvent window than more doped facets, with differences of approximately 0.4–0.5 V on both oxygen-terminated (2.79 V vs. 2.34 V) and hydrogen-terminated (4.04 V vs. 3.52 V) BDD surfaces. Additionally, more doped facets showed higher cathodic current densities at extreme potentials, indicating enhanced electrochemical activity toward hydrogen evolution. This narrowing directly impacts the signal-to-noise ratio and, consequently, the achievable LOD, particularly for analytes requiring high overpotentials. For phenolic compounds that require a wide potential window for oxidation, lower doping levels (10^19^–10^20^ cm^−3^) are therefore preferred, whereas heavy metal detection, which relies on stripping voltammetry, can tolerate moderate doping (10^20^–10^21^ cm^−3^) to benefit from enhanced conductivity. Quantitative comparisons of doping conditions and resulting performance across multiple studies are compiled in Table 1, where readers can directly compare different electrode architectures [16,17,31,45,48]. Thus, how to maintain the conductivity advantages of higher boron doping while suppressing the negative effects of potential window narrowing, background current increase, and impurity phase formation remains an open question.

Boron doping also affects selectivity through its influence on background current, peak separation, and the working potential window [16,24]. Yang et al. [24] found that the boron content influences the electrocatalytic current and response sensitivity of BDD electrodes, but rich crystal facets and low background current are equally important for achieving selective multi-analyte detection. Their results showed that BDD electrodes with a moderate boron doping level exhibited good peak separation in the simultaneous detection of dopamine and melatonin, indicating that moderate doping can maintain a high response current while keeping low background current and multi-component recognition capability.

Using heavy metal ion detection as an example, boron doping also affects the sensitivity, detection limit, and simultaneous recognition capability of BDD electrodes. As discussed above, Marton et al. [17] systematically investigated the influence of boron concentration, surface morphology, and structure of BDD films on the performance of Bi-modified BDD electrodes. Their results further confirmed that the active sites introduced by boron on the surface and subsurface increased significantly with increasing doping level, and the stripping peak currents of heavy metal ions became clearly distinguishable from undetectable. However, further increasing the doping level decreased the sensitivity, possibly due to increased *sp*^2^ phase content and reduced crystal quality.

The stepwise evolution of performance with increasing boron concentration follows a distinct non-monotonic pattern [17,54,55]. At low doping levels (<10^19^ cm^−3^), the limited carrier concentration results in poor conductivity and weak electrochemical responses, making most heavy metal ions undetectable [54,55]. As the doping level increases into the moderate range (10^19^–10^20^ cm^−3^), the carrier density and active site density rise substantially, and stripping peak currents for Zn^2+^, Cd^2+^, and Pb^2+^ become clearly distinguishable [17]. The optimal performance window is observed at approximately 10^20^–10^21^ cm^−3^, where sensitivity reaches its maximum (2.72–7.01 nA/nM). However, beyond this optimal range (>10^21^ cm^−3^), further boron incorporation induces lattice distortion, promotes the formation of non-diamond *sp*^2^ carbon phases at grain boundaries, and reduces crystal quality [15,21,56]. The consequence is a decline in sensitivity, an increase in background current, and a narrowing of the electrochemical window effects that collectively degrade the signal-to-noise ratio and compromise detection performance [21,56]. This non-monotonic behavior has been confirmed across multiple studies and underscores the necessity of identifying pollutant-specific optimal doping ranges rather than simply maximizing boron concentration [15,17].

In this way, the essence of boron doping regulation is to change the carrier concentration, grain size, grain boundary density, non-diamond phase content, and electrochemically active surface area by adjusting the incorporation level of boron atoms in the diamond lattice, thereby synergistically influencing the electrode conductivity, background current, and working potential window. Low doping limits electron transfer efficiency, while excessive doping may increase *sp*^2^ carbon content, narrow the potential window, and raise background current.

Quantitatively, the penalties associated with excessive doping include: a reduction in the electrochemical window by up to 0.8 V [31,55,57], an increase in background current by 2–3 times [31,57], and a decrease in sensitivity by approximately 30–50% beyond the optimal doping range for certain analytes [17]. These penalties arise from the formation of *sp*^2^ carbon phases at grain boundaries, which introduce additional electronic states that facilitate non-faradaic current and parasitic reactions [57]. Therefore, boron doping should be considered a “window-type” regulation strategy, and its optimal range needs to be determined based on electrode morphology, target pollutants, electrolyte environment, and detection mode [6,45,58].

### 3.2. Carbon Nanomaterial Modification: Surface Area and Conductive Network via Sensitivity and Detection Limit

**Figure 3:**
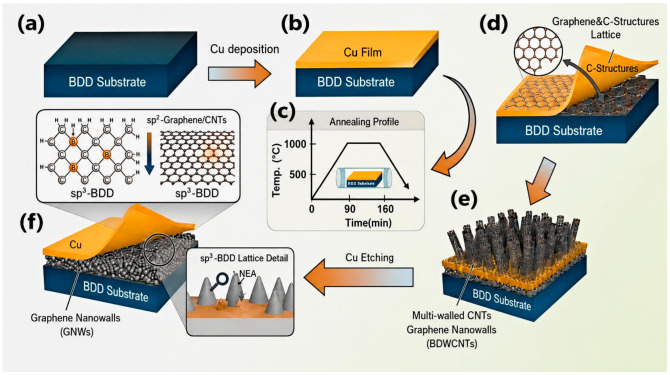
Fabrication route of carbon nanostructure-modified BDD electrodes. Original schematic prepared by the authors. (**a**) Bare BDD electrode; (**b**) surface activation/functionalization; (**c**–**f**) stepwise assembly of carbon nanomaterials.

The intrinsically inert surface of BDD electrodes, together with limited electrochemically active area and enrichment sites, often leads to low response currents and insufficient detection limits in trace analysis [52]. Therefore, constructing composite sensing interfaces by introducing carbon nanomaterials has become an important strategy to improve the sensitivity and lower the detection limit of BDD electrodes [18,19,59]. Figure 3 illustrates the preparation process of the carbon nanomaterial-modified BDD electrode. Carbon nanomaterials can enlarge the electroactive area, build conductive networks, and provide adsorption/enrichment sites, thereby improving the electrochemical reaction environment at the electrode solution interface [60].

The nanoscale characteristics of these materials are central to their sensing performance [60,61,62]. At the nanoscale, the surface-to-volume ratio increases dramatically, exposing a larger fraction of atoms at the surface and providing abundant active sites for target analyte interactions [62]. For carbon nanomaterials, this translates into three critical advantages over bulk carbon materials: (i) enhanced analyte adsorption due to high surface energy and abundant defect sites [63,64]; (ii) accelerated electron transfer kinetics due to shortened diffusion pathways and quantum confinement effects [64,65]; and (iii) improved sensitivity for trace-level detection due to efficient preconcentration of analytes at the nanoscale interface [65]. These size-dependent properties fundamentally distinguish nanomaterial-modified electrodes from their bulk counterparts and underpin the performance enhancements reported in the case studies discussed below [61]. Graphene, graphene oxide, reduced graphene oxide, carbon nanotubes (CNTs), graphene quantum dots, and carbon nanospheres have been widely used for highly sensitive electrochemical detection of environmental pollutants. Their advantages include enlarged electroactive surface area, enhanced electron transfer, additional adsorption sites, and improved sensing interface stability.

Carbon nanomaterials enhance BDD electrode performance through two main mechanisms. First, they significantly increase the effective active area, creating a richer reaction interface that promotes target analyte accumulation—particularly beneficial for heavy metal detection, where the pre-concentration step directly determines stripping peak currents [41,42,66]. Second, they build efficient conductive networks on the BDD surface, providing fast electron transfer pathways that reduce charge-transfer resistance and accelerate redox kinetics [41,67].

This configuration creates a synergy between the *sp*^2^ carbon in the modifier layer and the *sp*^3^ carbon of the BDD substrate [7,41,68]. The *sp*^2^ carbon network offers high electrical conductivity and abundant reactive sites for analyte adsorption and electron transfer [41,68,69], while the *sp*^3^ diamond backbone provides chemical inertness, a wide electrochemical window, and low background current [7,21]. The resulting hybrid interface combines the conductivity of a carbon nanostructure with the electrochemical stability of diamond, achieving both high sensitivity (from the *sp*^2^ network) and stable performance (from the *sp*^3^ support) [7,41,68]. Such synergy is particularly advantageous in complex water matrices where electrode fouling and corrosion are concerns, as the underlying diamond substrate maintains structural integrity and low background current even when the modifier layer is partially compromised [7,21].

Different carbon nanomaterials offer distinct advantages: graphene and its derivatives provide high electrical conductivity (~10^3^–10^4^ S/m) and two-dimensional electron transport pathways [41,61,63,69]; CNTs form one-dimensional conductive channels that promote charge transfer and mass diffusion [61,63,68]; carbon nanospheres contribute a high specific surface area (typically 500–1500 m^2^/g) and tunable pore structures for analyte enrichment [42]; functionalized carbon materials introduce surface functional groups (carboxyl, hydroxyl, and amino) that enhance the adsorption of heavy metal ions or organic pollutants [63,70,71]. Furthermore, the limitations of single materials can be overcome by combining multiple nanomaterials to achieve synergistic enhancement of the detection performance of BDD electrodes [41,67,69]. For example, the combination of CNTs and graphene can reduce the contact resistance and improve the charge transfer rate of the hybrid-modified BDD electrode [7,21,72].

**Figure 4:**
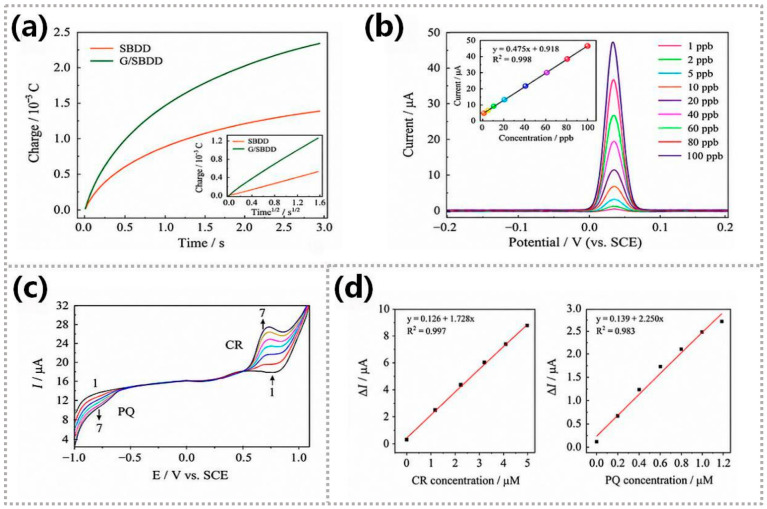
Carbon nanostructure-modified BDD electrodes for Pb^2+^ sensing. (**a**,**b**) Electroactive surface area and Pb^2+^ stripping response of G/SBDD electrodes. (Reprinted from Ref. [41] Copyright 2020, with permission from Elsevier.). (**c**,**d**) Differential pulse voltammetric responses and calibration plots of graphene-modified BDD electrodes for simultaneous detection of carbaryl and paraquat. (**c**) Differential pulse voltammograms recorded at the BDDGR electrode in acetic acid/sodium acetate buffer supporting electrolyte without analytes (trace 1) and with increasing concentrations of CR and PQ (traces 2-7): trace 2, 1 μM CR + 0.2 μM PQ; trace 3, 2 μM CR + 0.4 μM PQ; trace 4, 3 μM CR + 0.6 μM PQ; trace 5, 4 μM CR + 0.8 μM PQ; trace 6, 5 μM CR + 1.0 μM PQ; and trace 7, 6 μM CR + 1.2 μM PQ. Experimental parameters: sp = 6 mV, MA = 800 mV; potential window, −1.0 to +1.75 V vs. SCE. (**d**) Calibration plots of the peak currents measured at +0.74 V vs. SCE as a function of CR concentration and at −0.80 V vs. SCE as a function of PQ concentration. (Reprinted from Ref. [49]).

Taking heavy metal ion detection as an example, Pei et al. [41] prepared a self-supporting BDD electrode modified with graphene nanowalls (G/SBDD). They found that the graphene sensing layer increased the electrode’s active area, enhanced the response signal, and reduced charge-transfer resistance. The electrode showed a good linear response to Pb^2+^ in the range of 1–100 ppb, with a sensitivity of 0.475 μA·L·μg^−1^·cm^−2^ and a detection limit of 0.21 μg/L (Figure 4a,b), demonstrating that carbon nanomaterial modification can improve the sensitivity and low-concentration detection capability of BDD electrodes by optimizing the enrichment and stripping processes. Pop et al. [49] constructed a graphene-modified BDD electrode (BDDGR) for the simultaneous detection of carbaryl (CR) and paraquat (PQ). Compared with the unmodified BDD electrode, the BDDGR electrode exhibited a larger active surface area and an approximately four-fold higher useful signal toward CR oxidation, indicating the electrocatalytic effect of graphene on the BDD sensing interface. As shown in Figure 4c,d, the BDDGR electrode enabled simultaneous detection of CR and PQ in acetate buffer by differential pulse voltammetry, with linear ranges of 1–6 μM for CR and 0.2–1.2 μM for PQ. The corresponding sensitivities reached 33.27 μA·μM^−1^·cm^−2^ for CR and 31.83 μA·μM^−1^·cm^−2^ for PQ, with detection limits of 0.07 μM and 0.01 μM, respectively. These results indicate that graphene modification effectively enhances the sensitivity and low-concentration detection capability of BDD electrodes by enlarging the electroactive surface area and constructing efficient conductive pathways for interfacial charge transfer.

Beyond laboratory-scale sensing studies, BDD-based electrochemical platforms are being extended toward integrated water-quality monitoring systems [44,53,73]. A representative example involves graphene-modified BDD electrodes fabricated via hot-filament chemical vapor deposition, combined with anodic stripping voltammetry for the trace detection of Pb^2+^, Cd^2+^, Cu^2+^, and Zn^2+^ in aqueous environments [41,53,69]. Such a system integrates electrode fabrication, structural optimization, signal acquisition, and electrochemical analysis into a complete sensing chain [53,73]. These engineering-oriented studies further demonstrate that the practical performance of BDD sensors strongly depends on interfacial structure regulation, charge-transfer efficiency, anti-interference capability, and long-term operational stability [7,21,53].

However, carbon nanomaterial modification is not without limitations [18,74]. Because carbon modification layers are typically immobilized on the BDD surface by drop-casting, deposition, or composite coating, they may suffer from uneven dispersion, aggregation, insufficient adhesion, or detachment during long-term use [32]. In complex water matrices, organic matter, salt ions, and coexisting pollutants may also cover the carbon material surface, reducing the number of active sites and attenuating the detection signal [31,59]. Therefore, although carbon nanomaterial modification can effectively improve the sensitivity and detection limit of BDD electrodes, its interface stability, reproducibility, and long-term reliability still require further optimization.

As a result, the core role of carbon nanomaterial modification of BDD electrodes can be summarized as “increased surface area-conductive network construction-enhanced enrichment capability-lower detection limit” [41,42,68,69]. This strategy is primarily aimed at overcoming the insufficient sensitivity of BDD electrodes by improving the electrode interface reaction conditions through nanostructure modification. However, in practical applications, it is necessary to further balance the relationship between sensitivity enhancement and interface stability [7,21], and to improve the adhesion stability between the carbon nanomaterial modification layer and the BDD substrate by means of post-treatment curing, composite structure design, or controlled deposition techniques [14,41,67], thereby promoting the long-term reliable application of BDD electrodes in water pollutant detection [7,41,42].

### 3.3. Metal Nanomaterial Modification: Catalytic Activity and Charge Transfer via Selectivity

The relatively inert surface of BDD electrodes often leads to slow redox kinetics for some target pollutants on unmodified BDD, resulting in insufficient peak current responses or poor peak separation. Metal nanomaterials generally have a high specific surface area, good conductivity, and outstanding electrocatalytic activity [3,8,19,20,21]. For metal nanoparticles, the nanoscale size introduces unique catalytic properties that are absent in bulk metals [20,75]. As particle size decreases into the nanometer regime (typically 1–100 nm), the proportion of surface atoms with low coordination numbers increases significantly, creating more catalytically active sites [75]. Concurrently, the electronic structure of metal nanoparticles becomes size-dependent due to quantum confinement effects, which can lower the activation energy for redox reactions and enhance charge transfer kinetics [20]. Furthermore, the high specific surface area of metal nanoparticles, which is substantially larger than that of bulk metals, enables efficient analyte preconcentration through adsorption, complexation, or alloy formation at the nanoparticle surface, directly contributing to enhanced sensitivity and lowered detection limits [17,30,76].

Introducing them onto the BDD surface can provide highly active reaction sites while maintaining the low background current and stability of BDD [8,77], accelerating interfacial charge transfer and thus improving the detection selectivity for target pollutants. Related studies have also indicated that surface modification strategies using metal nanoparticles, nanocarbons, and polymers can further enhance the electroanalytical performance of BDD electrodes [18,32,78].

Metal nanomaterials improve BDD electrode performance through three mechanisms. First, the high surface area of dispersed metal nanoparticles creates numerous catalytic centers that facilitate analyte enrichment and adsorption [17,20,30]. Second, metals such as Au, Bi, Pt, and Ir provide good conductivity and catalytic activity, reducing kinetic barriers and enhancing peak currents [17,20,76]. Third, specific interfacial interactions between metal nanoparticles and target pollutants, including alloying, complexation, adsorption, and catalytic reduction, enhance detection selectivity [17,76,79].

**Figure 5:**
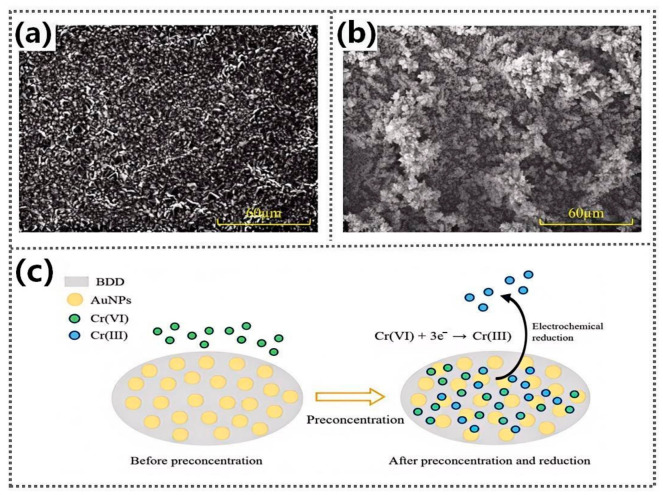
SEM images of (**a**) bare BDD electrode and (**b**) copper-modified BDD electrode (Reprinted from Ref. [80]). (**c**) Working principle of Cr(VI) preconcentration and electrochemical reduction on AuNPs-BDD electrode. Reprinted from Shellaiah and Sun [30], based on Xu et al. [76].

The improvement in selectivity through metal nanomaterial modification mainly stems from specific interfacial interactions between the metal and the target. Marton et al. [17] demonstrated that Bi-modified BDD electrodes combine the wide potential window and low background current of BDD with the enrichment effect of Bi for heavy metal ions such as Pb^2+^, Cd^2+^, and Zn^2+^, thereby enhancing the characteristic peak responses in anodic stripping voltammetry. Their simultaneous detection performance was influenced not only by the Bi modification layer but also by the boron doping level, surface morphology, and grain structure of the BDD substrate. Xu et al. [76] constructed an AuNP-modified BDD electrode for Cr(VI) detection. As illustrated in Figure 5c, AuNPs provide active sites for Cr(VI) preconcentration and facilitate its electrochemical reduction to Cr(III), thereby improving the reduction response of Cr(VI). The electrode achieved a detection limit of 1.19 μg/L with a linear range of 10–1000 μg/L. In addition to heavy metals, bifunctional BDD-based interfaces have also been developed for phosphate and nitrate sensing [80].

Representative examples include Bi-modified BDD electrodes for simultaneous detection of Zn^2+^, Cd^2+^, and Pb^2+^, achieving detection limits of 1.97 nM, 0.57 nM, and 0.51 nM, respectively [17], and AuNP-modified BDD electrodes for Cr(VI) detection with a detection limit of 1.19 μg/L and a linear range of 10–1000 μg/L [76]. These cases demonstrate that, unlike carbon nanomaterials, which mainly rely on surface area and conductive networks to improve sensitivity, metal nanomaterials offer advantages in enhancing the response intensity, peak resolution, and detection selectivity for specific pollutants through metal active sites that promote adsorption, deposition, or catalytic reduction.

However, metal nanomaterial-modified BDD electrodes also have limitations [81]. First, metal nanoparticles are easily affected by deposition conditions; their size, distribution density, and adhesion stability directly influence detection reproducibility [22]. Aggregation of nanoparticles may reduce the effective active area and even increase the background current. Second, some metal modification layers may suffer from dissolution, passivation, or surface contamination during long-term detection or in complex water environments, thus affecting detection stability. Third, while metal nanomaterials provide strong catalytic or enrichment effects for specific targets, this selectivity can also lead to a limited scope of application. For example, a metal modification layer suitable for detecting Cr(VI) or Pb^2+^/Cd^2+^ may not be applicable to other pollutant systems [76]. Therefore, the design of metal nanomaterial-modified BDD electrodes must comprehensively consider the target pollutant type, modification layer stability, detection environment, and electrode reusability.

As a result, the core role of metal nanomaterial modification of BDD electrodes can be summarized as “increased active sites-enhanced catalytic reaction-accelerated charge transfer-improved selective recognition” [17,20,30,76,79]. This strategy compensates for the insufficient surface reactivity of bare BDD electrodes and is particularly suitable for pollutant detection that requires catalytic reduction, catalytic oxidation, or specific metal enrichment [30,76,79,82]. Future research should further focus on the controllable preparation of metal nanoparticles on the BDD surface [23,76,79], interfacial adhesion stability, and anti-interference performance in complex water matrices [7,21,79], and through the combined regulation of metal nanomaterials with carbon nanomaterials [41,83], polymers, or surface pretreatment strategies, to better address the synergistic improvement of sensitivity, selectivity, and stability [20,21,30].

### 3.4. Comparison of the Limitations of Various Strategies

Table 2 compares the principles, advantages, and limitations of the three strategies. Boron doping optimizes substrate conductivity and potential window [7,14,15,16,21,30]; carbon nanomaterials enlarge the active area and build conductive networks [41,42,69]; metal nanomaterials introduce catalytic sites for enhanced selectivity [17,20,76,79]. However, the synergistic optimization of sensitivity, selectivity, and stability remains challenging, particularly in complex water environments where modification layers are prone to aggregation, detachment, or structural degradation [7,21,41,57]. Post-annealing treatment offers a pathway to address these issues by strengthening the bonding between the modification layer and the BDD substrate, stabilizing the interface structure, and enhancing long-term robustness [25,41,79]. Quantitative comparisons of doping conditions and resulting sensing performance across representative studies are compiled in Table 3, where readers can directly compare different electrode architectures. This is discussed in detail in Section 4.

## 4. Synergistic Effects of Post-Annealing Treatment

### 4.1. Influence of Annealing Parameters on the Structure and Properties of the Modified Layer

Annealing is essentially a heat treatment process that regulates the energy state inside and on the surface of materials. It is an important post-treatment method for controlling the microstructure and service performance of boron-doped diamond (BDD) modified layers [25,26]. Post-annealing has been widely employed as a fabrication step in BDD electrode preparation [25,28,79], with notable examples including the two-step annealing process for Au nanoparticle deposition on BDD [28] and the thermal stabilization of Cu-modified BDD electrodes for nitrate reduction [25]. However, while these studies demonstrate the effectiveness of annealing, the systematic relationships among annealing parameters (temperature, duration, atmosphere, heating rate), interfacial structural evolution, and sensing performance [25,26,27], and crucially, how annealing couples with doping and modification to achieve balanced sensitivity, selectivity, and stability, remain underexplored in the sensing literature. This section addresses this gap by treating annealing as a standalone regulatory strategy [6,19,21,30].

This section examines annealing from three perspectives: (Section 4.1) the influence of annealing parameters on the structure and properties of the modified layer, (Section 4.2) the effect of annealing on interfacial stability and long-term sensing behavior, and (Section 4.3) the multi-parameter coupling optimization logic that integrates annealing with doping and modification.

Its effects are mainly reflected in defect repair, surface termination adjustment, evolution of non-diamond carbon phases, and boron doping activation [8]. Previous studies have shown that BDD modified layers, after deposition or composite construction, usually contain a certain number of grain boundary defects, residual stress, and a small amount of *sp*^2^ carbon phase. These factors not only affect the electrical conductivity of the material but also further change its electrochemical window, interfacial charge transfer behavior, and long-term service stability [21,25,50]. Appropriate annealing can promote atomic rearrangement and structural reconstruction through thermal activation, thereby reducing residual stress and defect density, improving the intergranular bonding state and film compactness, and consequently enhancing the structural stability and electrical properties of the modified layer [18,25]. However, when the annealing temperature is too high or the holding time is too long, the surface layer may undergo graphitization, and boron elements may segregate or diffuse, leading to a decline in interfacial bonding strength and eventual deterioration of material properties [26,27,56]. Therefore, the influence of annealing parameters on BDD modified layers exhibits significant coupling effects and a process window [25,26,27].

Regarding the influence of annealing temperature, low-to-medium temperature annealing generally helps to remove surface-adsorbed impurities, residual organic matter, and weakly bound amorphous carbon, while releasing the internal stress accumulated during growth, with relatively limited damage to the dominant *sp*^3^ skeleton of diamond [7,21,27]. As the temperature increases, the atomic migration ability in grain boundaries and defect regions strengthens, and the local disordered structure can undergo reconstruction, thereby reducing carrier scattering and improving the effective activation degree of boron acceptors [26,54]. Macroscopically, this manifests as a decrease in resistivity and a reduction in charge-transfer resistance [25,27]. For electrochemical applications, this change is usually accompanied by improved background current stability and better electrode response repeatability [7,21]. However, when the annealing temperature is further increased, especially in high-defect-density or nanocrystalline BDD systems, the surface layer and grain boundary regions are more prone to the transition from *sp*^3^ to *sp*^2^, thus enhancing the graphitization tendency [27,52,56].

The evolution of the *sp*^2^/*sp*^3^ ratio as a function of annealing temperature follows a characteristic pattern. At temperatures below 400 °C, the *sp*^3^ framework remains largely intact, with only surface adsorbates and weakly bound amorphous carbon being removed [7,21]. Between 400 °C and 600 °C, the *sp*^2^/*sp*^3^ ratio begins to increase gradually as thermal energy enables the rearrangement of metastable carbon atoms at grain boundaries into more energetically favorable *sp*^2^ configurations [27,56]. Above 600 °C, particularly in highly boron-doped or nanocrystalline BDD, the transformation accelerates significantly, leading to pronounced surface graphitization and the formation of conductive *sp*^2^ networks along grain boundaries [27,56,57]. It should be noted, however, that these temperature thresholds are not absolute and depend strongly on the boron doping level and film microstructure. For instance, Zhao et al. [26] demonstrated that heavily boron-doped diamond films (h-BDD-2) remained structurally stable up to 800 °C, with graphitic signals appearing only at 850 °C, whereas undoped microcrystalline diamond films were almost completely oxidized at 800 °C. This confirms that the onset temperature for graphitization and oxidation is significantly elevated by heavy boron doping, highlighting the importance of considering boron concentration when applying the above temperature ranges.

Although an appropriate amount of *sp*^2^ phase can provide faster channels for electron transport and increase the apparent charge transfer kinetics, it simultaneously compresses the potential window of BDD, increases the background current, and weakens the chemical inertness of the material [21,56]. This duality represents a critical trade-off: the same *sp*^2^ network that enhances electron transfer also introduces additional electronic states that facilitate parasitic reactions and non-faradaic current, narrowing the electrochemical window and degrading the signal-to-noise ratio [56,57]. The optimal annealing condition, therefore, corresponds to a point where sufficient *sp*^2^ connectivity is achieved for efficient charge transport without excessive graphitization that would compromise the electrochemical stability and selectivity [27,56].

**Figure 6:**
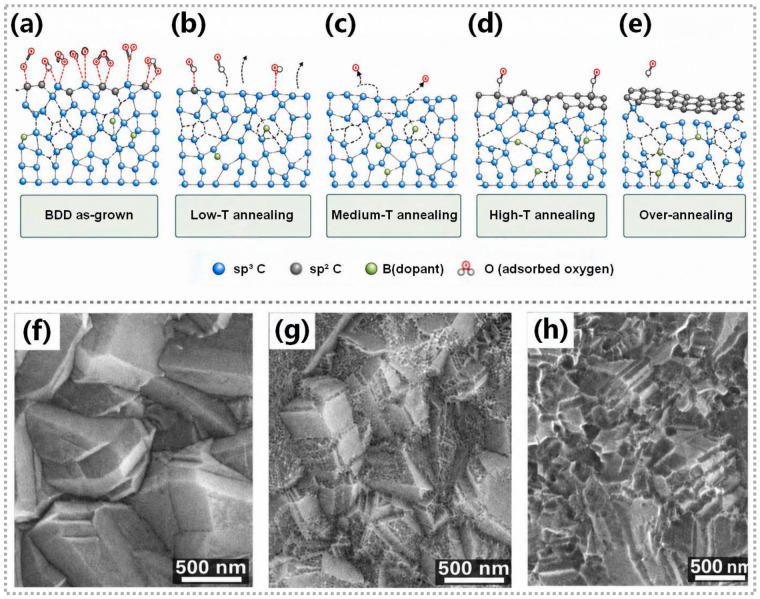
(**a**–**e**) Original schematic diagram of the temperature-dependent surface evolution mechanism of BDD under annealing. (**f**–**h**) SEM images after oxidation at 600 °C in air for (**f**) 10 min, (**g**) 30 min, and (**h**) 90 min. (Reprinted from Ref. [27]).

This dual effect is closely related to the thermally induced evolution of boron-related defects and surface carbon phases. As illustrated in Figure 6a–e, annealing temperature induces a stepwise evolution of the BDD surface, including the desorption of weakly adsorbed oxygen species, migration and rearrangement of surface defects, formation of local *sp*^2^ carbon domains, and eventual graphitization under over-annealing conditions. Figure 6f–h demonstrates that prolonged high-temperature oxidation progressively damages the BDD surface: shallow etching pits appear after 30 min, followed by severe grain degradation after 90 min [27]. Therefore, the regulation of BDD modified layers by annealing temperature presents an obvious “dual effect”: there exists an optimal range that balances the improvement of conductivity and the preservation of the intrinsic stability of diamond [26,27,56].

This dual effect can be quantified from the *sp*^2^/*sp*^3^ perspective: R1-2-3. It is generally recognized that *sp*^2^ carbon impurities influence the electrochemical window, background current, and charge-transfer kinetics [56]. When the *sp*^2^ content is low (i.e., the material is predominantly *sp*^3^), the electrode maintains a wide potential window and low background current. As the *sp*^2^ content increases to a moderate level, the conductive *sp*^2^ network can improve charge-transfer kinetics, reducing charge-transfer resistance and enhancing sensitivity. However, when the *sp*^2^ content becomes excessive, the potential window narrows significantly, and the background current rises, effectively negating the sensitivity gains and degrading selectivity [27]. The optimal balance between conductivity and electrochemical stability typically corresponds to a moderate sp^2^/sp^3^ ratio, where the conductive *sp*^2^ network provides enhanced electron transfer, while the sp^3^ diamond matrix maintains a sufficiently wide potential window and low background current [26,56].

The annealing atmosphere mainly determines the surface chemical state and the evolution path of non-diamond carbon phases. Under an inert atmosphere (e.g., Ar, N_2_), the annealing process largely avoids severe oxidation, so the heat treatment effects are mainly stress release, defect rearrangement, and local structural reconstruction [7,21,27]. In this atmosphere, the *sp*^2^/*sp*^3^ ratio tends to increase gradually with temperature as thermal energy promotes rehybridization at defect sites, but the overall carbon phase ratio remains largely determined by the intrinsic thermal stability of the diamond structure [52,56]. Under a reducing atmosphere (e.g., H_2_), hydrogen atoms preferentially terminate dangling bonds and etch non-diamond carbon phases, effectively removing pre-existing *sp*^2^ impurities and maintaining a cleaner *sp*^3^ surface [6,7,86].

Under an oxidizing atmosphere (e.g., air or O_2_), *sp*^2^ carbon is preferentially oxidized and removed at moderate temperatures (400–600 °C), effectively reducing the *sp*^2^/*sp*^3^ ratio; however, at higher temperatures, the oxidizing environment can also attack the diamond lattice, leading to surface etching and the formation of pits, as shown in Figure 6f–h [27]. This dual behavior underscores the importance of atmosphere selection: oxidizing annealing can purify the BDD surface by removing *sp*^2^ impurities, but the conditions must be carefully controlled to avoid etching of the diamond itself [27]. In contrast, vacuum annealing is generally more conducive to surface adsorbate desorption and structural rearrangement, but it is also often accompanied by a more pronounced tendency for surface graphitization, as the absence of any reactive gas allows carbon atoms at the surface to reorganize freely into thermodynamically stable *sp*^2^ configurations [41,47,52].

Vacuum annealing is generally more conducive to surface adsorbate desorption and structural rearrangement, but it is also often accompanied by a more pronounced tendency for surface graphitization [41,52,56]. In contrast, annealing in an oxidizing atmosphere can not only remove weakly stable amorphous carbon and some graphitic phases but also introduce oxygen-containing functional groups such as hydroxyl, carbonyl, and carboxyl groups on the BDD surface, forming an oxygen-terminated surface [7,87,88]. This process generally increases surface hydrophilicity and interfacial wettability, and has a positive impact on the kinetics of some electrochemical reactions [6,46,88]. However, if the oxidizing conditions are too strong, surface etching, roughening, or even local structural damage may occur, thereby reducing the integrity and long-term stability of the film [26,27]. Meanwhile, a reducing atmosphere, especially H_2_ annealing, is often used to restore or maintain a hydrogen-terminated surface [6,86]. Hydrogen-terminated BDD typically exhibits high surface conductivity and an energy band structure different from that of oxygen-terminated BDD, thus offering unique advantages in field emission, electrochemical sensing, and interfacial electron transfer [7,8,86]. It can be seen that the annealing atmosphere not only affects the carbon phase ratio of the BDD modified layer but also profoundly determines its surface termination type and interfacial reaction characteristics [6,86,88].

In addition to temperature and atmosphere, annealing time is also an important parameter influencing the structural evolution of BDD modified layers. Under specific temperature and atmosphere conditions, too short an annealing time is often insufficient to complete impurity desorption, stress release, and defect reconstruction, so the improvement in material properties is limited [25,27]. As the time increases, structural rearrangement gradually becomes sufficient, and the crystalline quality, interfacial bonding state, and electrical properties of the modified layer are generally further optimized [25,89]. However, when the annealing time is too long, grain coarsening, increased surface roughness, *sp*^2^ phase enrichment, and enhanced interfacial diffusion may occur. Especially under high-temperature oxidizing atmospheres, excessively long annealing may also cause surface etching and film thinning, thereby reducing electrode stability [26,27].

**Figure 7:**
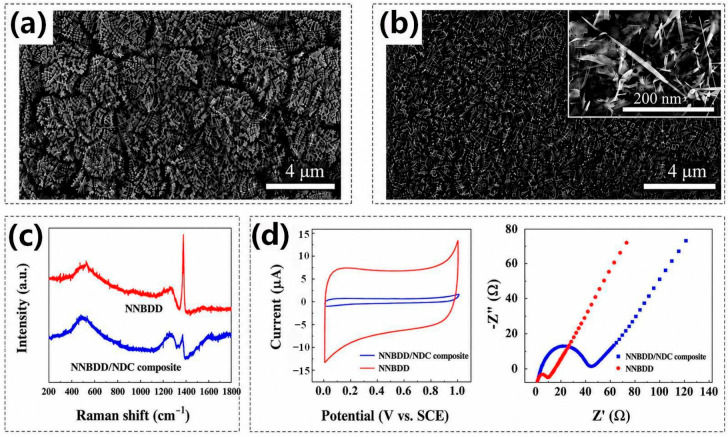
Annealing-induced structural and electrochemical evolution of NNBDD-based electrodes. (**a**,**b**) SEM images showing the surface morphologies of NNBDD and the NNBDD/NDC composite, with the inset in (**b**) presenting a high-magnification view of the nanoscale carbon structure. (**c**) Raman spectra of NNBDD and the NNBDD/NDC composite, showing the structural changes associated with non-diamond carbon species after annealing. (**d**) Cyclic voltammetry and electrochemical impedance spectroscopy responses of NNBDD and the NNBDD/NDC composite, revealing the influence of annealing-induced surface reconstruction on electrochemically active area and interfacial charge-transfer behavior. (Reprinted from Ref. [89]).

From the perspective of structure-performance relationships, the influence of annealing on BDD modified layers is ultimately reflected in changes in electrical and electrochemical characteristics. First, moderate annealing helps to improve the activation efficiency of boron doping, reduce grain boundary barriers, and improve film continuity, which generally manifests as lower resistivity and better charge transport capability [25,26,54]. Second, changes in surface termination state and *sp*^2^/*sp*^3^ ratio significantly affect the electrochemical window, electrical double-layer behavior, background current, and electrocatalytic response to target molecules [6,21,56]. Generally speaking, BDD systems with high *sp*^3^ content and stable surface structure are more suitable for scenarios requiring a wide potential window, low background current, and high corrosion resistance [7,21]. In contrast, BDD with an appropriate amount of *sp*^2^ conductive network or specific surface terminal regulation is more conducive to accelerating electron exchange processes and improving the sensitivity of certain electrocatalytic or sensing reactions [46,47,56]. The optimization of annealing parameters is not simply about pursuing higher conductivity, but rather about achieving a comprehensive balance between the intrinsic stability of diamond, the interfacial electron transfer rate, and the target functional requirements [6,7,21]. Figure 7 further confirms this structure-performance relationship. After annealing, the non-diamond carbon phase is reduced, and the NNBDD morphology becomes clearer (Figure 7a,b), while the CV and EIS results show improved electrochemical activity and charge-transfer behavior (Figure 7c,d) [89]. This indicates that appropriate annealing can optimize both the surface structure and the electrochemical interface of BDD electrodes [89,90].

### 4.2. Effect of Annealing on Interfacial Stability and Long-Term Sensing Behavior

**Figure 8:**
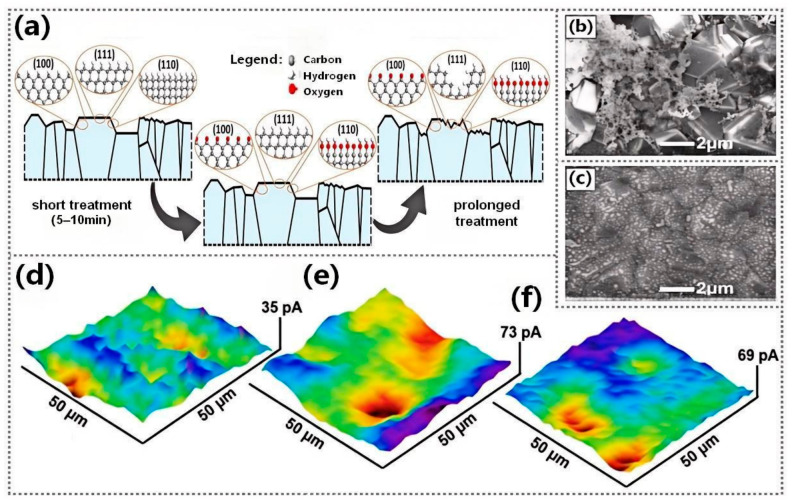
(**a**) Schematic visualization of BDD surface oxidation during high-temperature treatment in air at 600 °C (Reprinted from Ref. [27]). (**b**,**c**) SEM morphologies of Pt-BDD before and after annealing. (Reprinted from Ref. [91]) (**d**–**f**) SECM maps of heavily doped BDD after oxidation at 600 °C for different durations. (**d**) 10 min, (**e**) 30 min, and (**f**) 90 min (Reprinted from Ref. [27]).

Residual stress is usually introduced during the deposition of BDD thin films, accompanied by a certain degree of grain boundary defects and film-substrate interfacial discontinuity, especially in polycrystalline BDD and heteroepitaxial substrate systems. Appropriate annealing can promote local atomic diffusion and structural rearrangement, release internal stress, and reduce interfacial defect density, thereby improving film adhesion stability and reducing performance degradation caused by cracking, delamination, and local failure during long-term electrochemical operation [25,26,27]. Figure 8b,c shows the differences in surface morphology of Pt-BDD before and after annealing, supporting the conclusion that thermal annealing improves Pt growth quality and strengthens the interaction between Pt particles and the BDD surface. For continuous monitoring sensors, this enhanced interfacial stability is essential for maintaining active Pt sites, reducing modifier loss, and achieving stable sensing responses over repeated use [91].

In addition, annealing can significantly regulate the surface termination state and functional group distribution of BDD, thereby affecting the charge transfer process and adsorption behavior at the electrode/electrolyte interface. The surface of BDD can present hydrogen-terminated, oxygen-terminated, or mixed-termination states [86], each corresponding to different surface work functions, wettability, and interfacial reactivity. During annealing under vacuum, inert, or oxidizing atmospheres, the surface may undergo de-hydrogenation, oxidation, reconstruction, and functional group redistribution [88], thereby changing the background current, electrical double-layer structure, and adsorption/desorption kinetics of target analytes [88]. Therefore, annealing not only improves structural stability but is also an important way to regulate the surface chemical properties of BDD, with its effects closely related to specific process conditions.

In long-term sensing applications, appropriately annealed BDD electrodes generally exhibit lower baseline drift, better cycling stability, and higher signal repeatability [7,25,79]. However, excessive thermal oxidation may produce the opposite effect [26,27]. As shown in Figure 8a, high-temperature treatment in air induces time-dependent oxidation of the BDD surface. Short treatment mainly changes surface termination, whereas prolonged exposure leads to heterogeneous oxidation, etch-pit formation, and irreversible structural defects. The SECM maps in Figure 8d–f further show non-uniform local charge-transfer activity after oxidation at 600 °C, indicating that annealing has a parameter-dependent effect on interfacial stability. Therefore, annealing conditions should be carefully optimized to stabilize the sensing interface without damaging the BDD microstructure or reducing long-term electrochemical performance [27]. This is mainly attributed to the reduction in interfacial trap states, the homogenization of surface states, and the reduction in contamination-induced signal changes after annealing [6,7,27]. In scenarios such as electrochemical sensing, biosensing, and environmental monitoring, sensors often need to operate for long periods in complex media, so the stability of the interfacial state directly affects their service life and quantitative analysis accuracy [19,21,31]. It can be seen that annealing is an important post-treatment measure to improve the long-term stability of BDD sensors [25,27,79].

The improvement of BDD performance by annealing is not unidirectional; there is an obvious process window [26,27,56]. If the annealing temperature is too high, the holding time is too long, or the atmosphere is improperly controlled, it may cause local graphitization, excessive oxidation, and changes in the boron doping state on the BDD surface, leading to degradation of the conductive network, increased sheet resistance, and hindered charge transfer, ultimately manifested as decreased sensitivity, increased noise, and intensified signal drift [27,56]. Especially for highly boron-doped BDD, heat treatment may also change the local coordination state of boron and the carrier distribution, causing irreversible changes in electrochemical performance [16,26,27]. Therefore, the annealing process for BDD must achieve a balance among “defect repair, surface reconstruction, and electrochemical activity retention”, and the temperature, time, and atmosphere should be optimized according to the specific application requirements [25,26,27].

As a result, annealing has a significant impact on the interfacial stability and long-term sensing behavior of BDD. Moderate annealing helps to improve interfacial structural integrity, enhance surface chemical stability, and increase long-term monitoring consistency, while excessive annealing may lead to surface degradation and deterioration of electrical properties [25,27,79]. Future research can combine in situ characterization and long-term aging tests to further reveal the interfacial evolution mechanism of BDD under different annealing conditions, providing a basis for the synergistic optimization of high stability and high sensitivity [19,27,88].

### 4.3. Multi-Parameter Coupling Optimization Logic of Doping, Modification, and Annealing

The performance optimization of BDD electrodes is usually not determined by a single factor, but by the coupled effect of multiple parameters, including doping, surface modification, and annealing [6,7,19,21]. Among them, the doping level mainly controls the intrinsic conductivity, carrier concentration, and electrochemical activity of BDD, and is the primary factor determining the basic performance of the electrode [6,15,29,54]. Surface modification introduces specific functional groups, catalytic sites, or biological recognition units to adjust the interfacial selectivity, reaction kinetics, and anti-fouling ability, thereby expanding the application range of BDD in sensing, catalysis, and bioanalysis [6,19,30,82,87]. Annealing treatment further improves the long-term stability and signal consistency of the material by releasing residual stress, reconstructing the surface termination state, and optimizing the interfacial defect distribution [25,26,27,79]. These three factors are not simply additive; they exhibit significant coupling effects: the doping level determines the structural basis that can be modified and annealed; surface modification affects the interfacial reconstruction behavior during heat treatment; and annealing may in turn change the bonding mode, stability, and electrochemical response pattern of the modified layer [7,19,28,29,79,90].

In the actual optimization process, the doping concentration window should first be determined according to the application goal [6,29,54]. A lower boron doping level is conducive to maintaining high electrochemical stability, but the conductivity and electron transport capability may be limited [6,54]. Although excessively high doping can reduce resistance and enhance electrode activity, it is prone to cause a decline in crystal quality, an increase in non-diamond phases, and deterioration of long-term stability [27]. On this basis, surface modification is usually used as a functionalization means to compensate for the insufficient intrinsic selectivity of BDD [19,21,82]. However, the thickness, bonding strength, and interfacial uniformity of the modification layer directly affect the thermal stability and interfacial compatibility during subsequent annealing [28,87,90]. Therefore, modification strategies must take into account both functional introduction and thermal stability maintenance, avoiding performance degradation due to coating detachment, structural rearrangement, or interfacial mismatch [19,29,79,90,92].

Annealing is often regarded as a key regulatory step to optimize the overall stability of the system [25,26,27]. Appropriate annealing not only improves the bulk and interfacial defect states of BDD but also enhances the bonding stability between the modification layer and the substrate [28,79]. However, improper parameter settings may lead to degradation of the modification layer, loss of functional groups, or even interfacial inactivation [19,27,29]. Therefore, the optimization logic of “doping → modification → annealing” should follow the principle of synergistic design: “doping first, then modification, then annealing”. That is, doping regulates the intrinsic electrical properties, modification imparts target recognition or catalytic functions, and annealing achieves structural and interfacial stabilization, ultimately realizing a balance among sensitivity, selectivity, and lifetime [6,19,21,25,28,29].

In this way, the high-performance construction of BDD electrodes should shift from single-factor optimization to multi-parameter synergistic regulation [6,7,19,21]. By systematically studying the coupling relationships among doping concentration, modification type, and annealing conditions, a process-structure-performance mapping for specific application scenarios can be established, providing a theoretical and process basis for the design of highly stable and highly sensitive BDD devices [15,19,25,27,54].

The following section (Section 5) will examine how this triple strategy—doping, modification, and annealing—has been applied (or has been overlooked) in representative studies on the detection of different water pollutants.

## 5. Representative Case Studies for Different Pollutants

The previous sections have proposed a triple strategy, i.e., doping, modification, and annealing, to overcome the sensitivity-selectivity-stability triangle bottleneck [93]. Section 4 highlighted post-annealing as an important synergistic step that locks modified layers and stabilizes interfaces. This section examines how this triple strategy has been applied (or has been overlooked) in representative studies on the detection of heavy metal ions, phenolic compounds, and antibiotics/emerging contaminants.

### 5.1. Heavy Metal Ions

X. Yu et al. [41] pre-deposited a Cu film on self-supported BDD and annealed at 1000 °C in vacuum to catalytically grow an in situ graphene recognition layer. The resulting G/SBDD electrode was used for Pb^2+^ detection in seawater. It exhibited a linear range of 1–100 ppb, a sensitivity of 0.475 μA·L·μg^−1^·cm^−2^, a detection limit (LOD) of 0.21 μg/L, and a relative standard deviation (RSD) of 2.7%. Notably, it retained ~96% of its initial response after 60 days in seawater. This work demonstrates that annealing-induced graphene/BDD interfaces offer both high sensitivity and long-term stability in complex matrices. A systematic comparison of representative heavy metal ion sensors is provided in Table 3.

In another study, Yuan et al. [89] fabricated nanoneedle boron-doped diamond (NNBDD) composite films and then annealed at 800 °C in air to remove non-diamond carbon phases. The annealed NNBDD electrode was used for Pb^2+^ detection, showing a linear range of 1–80 μg/L and an LOD of 0.32 μg/L. Air annealing effectively eliminated *sp*^2^ carbon impurities, reduced background current, and stabilized the nanoneedle structure. Compared with the previous work, this work used a simpler annealing setup (air instead of vacuum) and still achieved a satisfactory LOD, suggesting that even mild oxidative annealing can be beneficial if the diamond surface is not over-etched.

Kim et al. [53] systematically optimized the substrate pretreatment and hot-filament chemical vapor deposition (HFCVD) conditions for BDD electrode fabrication. The optimized BDD electrode with a boron doping concentration of 8000 ppm showed high accuracy and precision in detecting Cd(II), Pb(II), and Cu(II) ions, with detection limits of 0.55 ± 0.05 μg/L for Cd(II), 0.43 ± 0.04 μg/L for Pb(II), and 0.74 ± 0.06 μg/L for Cu(II) in real water samples. The electrode exhibited excellent selectivity and repeatability, and when applied to real water samples, it demonstrated high accuracy without interference from various coexisting heavy metal ions. This work emphasizes the importance of deposition condition optimization—particularly the boron doping level—in achieving high-performance BDD sensors for multi-heavy-metal detection, and highlights the potential of unmodified BDD electrodes for practical applications when the doping concentration is carefully controlled.

Morris et al. [94] developed a vibrating boron-doped diamond electrode device for the analysis of ultralow concentrations of Cd(II) in water. By employing mechanical vibration of the electrode during anodic stripping voltammetry (SWASV), the mass transport efficiency was significantly enhanced, leading to excellent analytical performance without the need for surface modification or regular electrode replacement or polishing. The vibrating BDD electrode showed two linear response regions of 0.01–1 μg/L and 1–30 μg/L, with a detection limit of 0.04 μg/L and a quantification limit of 0.12 μg/L. This work shows that physical engineering alone can yield competitive sensitivity. The vibrating BDD electrode approach offers a notable advantage: it eliminates the complexity and stability concerns associated with modification layers while achieving high sensitivity and durability. This work demonstrates that physical engineering of the electrode, rather than chemical modification, can be a viable strategy for improving detection performance in certain applications.

Taken together, these four heavy-metal studies achieved low-μg/L or sub-μg/L LODs with good stability through optimized boron doping, physical mass-transport enhancement, and post-annealing strategies. The G/SBDD electrode showed superior long-term stability (96% after 60 days), likely due to the protective graphene layer. None of these four studies systematically optimized annealing parameters (temperature, time, atmosphere), leaving room for further improvement.

**Table 3:** Summary of BDD-based electrochemical sensors for heavy metal ion detection.

Electrode Type	B Doping Conditions	Modifier	Annealing Conditions	Detection Method	Target Analyte	Linear Range	LOD	Sensitivity	Stability	Real Sample	Ref.
G/SBDD	Self-supported BDD, B/C ratio not specified	In situ graphene (Cu-catalyzed)	1000 °C, vacuum	DPASV	Pb^2+^	1–100 μg L^−1^	0.21 μg/L	0.475 μA·L·μg^−1^ cm^−2^	~96% after 60 days in seawater	Simulated seawater (3.5 wt% NaCl)	Pei et al. 2020 [41]
CNs/BDD	B doping concentration = 1 × 10^19^ cm^−3^	Carbon nanospheres (drop-casting)	None	SWASV	Pb^2+^	1–140 μg L^−1^	0.01 μg/L	93 nA·L·μg^−1^	Not reported	Authentic/real water samples	Wang et al. 2024 [42]
Bi/BDD	1% CH_4_/H_2_, 10,000 ppm B/C	Bi film (in situ plating)	None	SWASV	Zn^2+^, Cd^2+^, Pb^2+^	1–10 μg L^−1^	Zn^2+^: 1.97 nM; Cd^2+^: 0.57 nM; Pb^2+^: 0.51 nM	Zn^2+^: 2.72 nA/nM; Cd^2+^: 3.81 nA/nM; Pb^2+^: 7.01 nA/nM	Not reported	Not reported	Marton et al. 2019 [17]
AuNPs/BDD	BDD film (details not specified)	Au nanoparticles (electrodeposition)	None	CSV	Cr(VI)	10–1000 μg L^−1^	1.19 μg/L	3.75 μA·L·mg^−1^	Not reported	Tap water	Xu et al. 2020 [76]
NNBDD	Nanoneedle BDD	None (nanostructured)	800 °C, air	DPASV	Pb^2+^	1–80 μg L^−1^	0.32 μg/L	Not reported	Not reported	Not reported	Yuan et al. 2023 [89]
BDD (high-durability)	BDD film (boron concentration optimized)	None	Not specified	DPASV	Heavy metals	1–1000 μg L^−1^	Cd(II): 0.55 ± 0.05 μg/L; Pb(II): 0.43 ± 0.04 μg/L; Cu(II): 0.74 ± 0.06 μg/L	Not specified	High durability reported	Daegu Seongseo river water	Kim et al. 2023 [53]
BDD (micro/nanocrystalline)	Micro/nanocrystalline BDD	None	None	SWASV	Pb^2+^	1–10 μg L^−1^	0.57 μg/L for BDND 2000	1.57 μA·L·μg^−1^· for BDND 2000	Not reported	Not reported	Arantes et al. 2014 [95]

Abbreviations: SWASV, square-wave anodic stripping voltammetry; DPASV, differential pulse anodic stripping voltammetry; CSV, cathodic stripping voltammetry; LOD, limit of detection.

### 5.2. Phenolic Compounds

During the growth of BDD under high temperature and boron-rich conditions, local *sp*^3^-carbon transformed into *sp*^2^ graphitic phases, forming a nanographite/BDD (NG/BDD) composite interface. This in situ graphitization can be viewed as a form of “growth-annealing”. The NG/BDD electrode was used for acetaminophen detection, achieving a linear range of 0.02–50 μM, an LOD of 5 nM, with good stability and reproducibility [47]. This case shows that high-temperature-induced *sp*^2^/*sp*^3^ reconstruction can create a highly conductive and stable interface for phenolic sensing without a separate post-annealing step. Representative organic pollutant sensors are summarized in Table 4.

A different approach was taken by Shi et al. [9], who fabricated nanostructured BDD (NBDD) electrodes by bias-assisted hot-filament CVD without any post-annealing. The NBDD electrode was used for phenol oxidation in simulated wastewater, showing significantly higher electrocatalytic activity than planar BDD and maintaining stable performance. While the absence of annealing did not prevent good stability (thanks to the robust nanostructure), the lack of a modified layer means that sensitivity is limited by the intrinsic BDD surface.

Pereira et al. [48] developed an electrochemical method for bisphenol A (BPA) determination using an unmodified boron-doped diamond electrode with differential pulse voltammetry (DPV). The sensitivity of the DPV measurements was significantly improved by using a predominantly hydrogen-terminated BDD electrode obtained by cathodic pretreatment. A highly linear analytical curve was obtained for BPA determination in the range of 0.44–5.2 μM, with quantification and detection limits of 0.71 μM and 0.21 μM, respectively. The method was successfully applied to monitor BPA concentration during electrooxidation in a flow reactor with an Nb/BDD anode, and no interference was observed from coexisting substances. This work is particularly notable because it demonstrates that even unmodified BDD electrodes—when properly pretreated to achieve hydrogen termination—can achieve satisfactory performance for phenolic compound detection, offering a simpler and more reproducible alternative to modified electrodes.

For phenolic compounds, high-temperature growth is thus effectively integrated with annealing into fabrication [9,47]. For nanostructured but unmodified BDD, annealing is not required [9]. However, for modified BDD intended for phenolic detection, post-annealing remains underexplored and could be a valuable future direction [19,21,25].

### 5.3. Antibiotics and Emerging Contaminants

Treetepvijit et al. [97] implanted Ni into BDD films and then annealed at 850 °C in H_2_ atmosphere for 10 min. Compared with the as-deposited BDD, the Ni-implanted and annealed electrode showed a sharper oxidation peak and higher peak current for tetracycline. In a flow-injection analysis system, it exhibited a linear range of 1.0–100 μM and an LOD of 10 nM. This work highlights that metal ion implantation combined with post-annealing can effectively activate the BDD surface for antibiotic detection. Studies employing annealing as a key fabrication step are compiled in Table 5.

Matsunaga et al. [46] investigated the effect of chemical surface termination on the electrochemical characteristics of boron-doped diamond powder (BDDP) for ciprofloxacin (CIP) detection. They prepared oxygen-terminated BDDP (O-BDDP) and hydrogen-terminated BDDP (H-BDDP), and mixed them with an insulating polyester resin binder to fabricate screen-printed diamond electrodes. The study revealed that surface termination significantly influences the electrochemical response toward CIP, with the optimized electrode configuration enabling sensitive detection. This work highlights that surface termination engineering—a parameter often overlooked in BDD sensor design—can be as critical as bulk doping or nanomaterial modification in determining sensing performance, particularly for organic pollutant detection, where adsorption and electron transfer kinetics are surface-sensitive.

Negrea et al. [70] designed a novel electrochemical sensor by modifying a commercial BDD electrode with graphene oxide (GO) reduced electrochemically and further decorated with silver (Ag), denoted as the Ag/GO/BDD electrode. Among a series of electrodes (BDD, GO/BDD, Ag/BDD, and Ag/GO/BDD), the Ag/GO/BDD electrode exhibited the best performance for tetracycline (TC) detection. Using square-wave voltammetry (SWV) operated at a step potential of 5 mV, modulation amplitude of 200 mV, and frequency of 10 Hz in alkaline medium, the electrode achieved a sensitivity of 46.6 μA·μM^−1^·cm^−2^ and a limit of detection of 5 nM. While the alkaline electrolyte-based procedure was limited for water monitoring due to chloride interference, the use of 0.1 M Na_2_SO_4_ supporting electrolyte eliminated this interference, enabling practical detection of TC in water samples. This work demonstrates the synergistic benefits of combining carbon nanomaterials (graphene oxide) with metal nanoparticles (silver) on a BDD substrate—a hybrid modification strategy that effectively integrates two of the three regulatory approaches discussed in this review.

For emerging persistent organic pollutants, Yuan et al. [28] deposited gold nanoparticles on BDD by two cycles of sputtering and annealing, forming a stable AuNPs/BDD interface. An aptamer was then immobilized for the detection of PCB-77. The sensor achieved an extremely low LOD of 0.32 fM. This case demonstrates that annealing-assisted metal nanoparticle decoration not only stabilizes the interface but also provides a reliable platform for aptamer-based ultrasensitive detection.

In this way, both antibiotic and emerging-contaminant studies used post-annealing (H_2_ or two-step annealing) and achieved low detection limits (10 nM for tetracycline, 0.32 fM for PCB-77) [28,97]. The AuNPs/BDD aptasensor is particularly impressive, showing that annealing can enable ultratrace detection by stabilizing the metal-diamond interface and allowing subsequent biofunctionalization [28,98]. While pesticide detection using BDD electrodes has been explored in recent studies, including screen-printed BDD platforms for carbaryl and paraquat detection [49], the limited number of systematic investigations precludes a dedicated case study in this review. Future work should focus on extending the triple strategy to pesticide sensing applications.

### 5.4. Bridging the Gap: How Annealing Integrates with Doping and Modification

The eleven case studies above reveal a clear pattern. In heavy metal detection, annealing (vacuum or air) helped form stable graphene interfaces or remove *sp*^2^ impurities. In phenolic sensing, high-temperature growth conditions (in situ annealing) or nanostructuring provided enhanced performance, but separate post-annealing is rarely applied. For antibiotics and emerging contaminants, annealing after metal deposition or ion implantation significantly improved peak resolution and lowered detection limits [99].

However, most of these studies applied annealing in an “ad hoc” manner without systematic optimization. None of them explored the coupling between doping level, modification type, and annealing parameters—the core of our proposed triple strategy. This gap directly leads to the future directions discussed in Section 6, such as multi-parameter optimization and AI-assisted screening.

Among the eleven case studies examined, only Pei et al. [41] (G/SBDD), Yuan et al. (NNBDD), and Yuan et al. [28] (AuNPs/BDD aptasensor) incorporated annealing as a deliberate fabrication step, and none systematically optimized annealing parameters in conjunction with doping level and modifier selection. This gap is particularly evident in the organic pollutant case studies, where post-annealing was either absent or applied without systematic optimization. The Ag/GO/BDD work by Negrea et al. [70] comes closest to a multi-strategy approach by combining carbon and metal modifications, yet annealing was not employed—suggesting that even hybrid modification strategies could benefit from thermal stabilization.

In particular, the combination of optimal boron doping (Section 3.1), a rationally chosen modifier (Section 3.2 and Section 3.3), and a carefully designed annealing step (Section 4) remains largely unexplored. We encourage researchers to adopt this integrated framework in future work.

## 6. Comprehensive Evaluation and Future Directions

### 6.1. Trade-Offs and Synergies Among Doping, Modification, and Annealing

Through the systematic summary of doping, surface modification, and annealing strategies, it is evident that improving the performance of BDD sensors often involves complex trade-offs among physicochemical parameters.

Conductivity vs. potential window. Increasing the boron doping concentration enhances electrical conductivity and reduces charge-transfer resistance, thereby improving sensitivity. However, excessively high doping introduces *sp*^2^ carbon impurities, increases background current, and narrows the electrochemical window, compromising selectivity in complex water matrices [15,16,17].

Sensitivity vs. interface stability. Modifying the BDD surface with carbon nanomaterials or metal nanoparticles significantly increases the density of active sites and catalytic activity. However, if such modification layers rely only on physical adsorption, they are prone to aggregation or detachment during long-term operation or at high potentials, severely affecting electrode lifetime [18,32,78].

Annealing is a double-edged sword. Post-annealing treatment is an important means to strengthen interfacial adhesion and activate dopants. However, if the annealing temperature is too high or the atmosphere is improperly controlled, it may cause excessive sintering of nanoparticles or oxidative etching of the diamond surface, damaging the intrinsic electrochemical activity of the sensor [25,26,27].

As a result, the design of BDD sensors cannot pursue extreme values of a single metric in isolation. Instead, the optimal balance should be sought within the integrated framework of doping, modification, and annealing to overcome the sensitivity-selectivity-stability triangle bottleneck.

### 6.2. Future Directions

Looking forward, several promising directions can be identified to further advance BDD-based electrochemical sensors for water pollutant detection. These include multi-scale composite design, AI-assisted high-throughput screening, real-world validation, dual-function platforms that integrate sensing and remediation, and multi-parameter sensing systems. Each is discussed below.

First, future research should shift from single-material modification to multi-scale composite design. For example, combining the high conductivity of carbon nanomaterials with the high catalytic activity of metals or metal oxides to construct three-dimensional heterostructures can achieve simultaneous optimization of charge transport and catalytic response [100]. Future efforts may further extend the in situ growth strategy by exploring alternative catalytic metals, such as Ni and Co, for graphene formation on SBDD electrodes, thereby improving heavy-metal sensing performance through enhanced interfacial conductivity and structural stability. More broadly, strengthening the interaction between the modification layer and the BDD substrate through in situ growth, chemical bonding, or interface-engineering strategies represents a key route to overcoming the long-term stability limitations of modified BDD electrodes.

Second, given the vast parameter space of BDD structural regulation (doping concentration, modifier composition, annealing temperature/time/atmosphere), introducing artificial intelligence (AI)-assisted design will become an important trend. Machine learning algorithms can model large datasets of structure and performance, efficiently screening optimal configurations from multi-dimensional variables, thereby enabling customized development of sensor performance. The development of non-destructive quality assessment methods (e.g., X-ray diffraction-based mapping of stress, grain orientation, and *sp*^3^/*sp*^2^ ratio) could provide valuable feedback for optimizing deposition parameters and ensuring batch reproducibility of BDD electrodes.

Specific machine learning approaches—such as random forest regression for predicting sensitivity from synthesis parameters, Gaussian process regression for uncertainty quantification in performance prediction, and Bayesian optimization for efficient exploration of the high-dimensional parameter space—are particularly promising for BDD sensor development [101,102]. The key challenges for implementing AI-assisted design in this field include: (i) the generation of sufficiently large, standardized datasets through systematic experimental design, (ii) the development of consistent reporting protocols that enable data sharing across laboratories, and (iii) the integration of machine learning predictions with experimental validation in an iterative optimization loop.

Third, most current BDD sensors have demonstrated excellent performance only in ideal laboratory electrolyte environments. Future work needs to intensify validation in real matrices such as industrial wastewater and surface water. To address complex organic interference and fouling in real water, BDD electrodes with self-cleaning capabilities or specific anti-fouling coatings (e.g., polymer films) should be developed to ensure long-term reliability for on-site, online monitoring [103]. Beyond real-water validation, future BDD sensors should further move toward integrated platforms for harsh-environment monitoring. Biomimetic protective coatings, such as fish-scale-inspired structures, may improve the anti-corrosion and anti-erosion durability of BDD-based sensing interfaces under high-temperature, high-pressure, or high-flow conditions. When coupled with digital monitoring and data-traceability strategies, such systems could enhance the reliability and practical deployability of BDD electrodes in complex industrial scenarios.

Fourth, beyond standalone sensing, BDD electrodes offer a unique opportunity for integrated detection and degradation of water pollutants. Because BDD exhibits both excellent electrochemical sensing capabilities and strong oxidative power for organic mineralization, the same electrode can potentially be used to first detect a pollutant and then electrochemically degrade it [104]. This concept, sometimes referred to as “sense-and-treat” or “electrochemical advanced oxidation process (EAOP) with online monitoring”, has been demonstrated in a few studies. For example, Vasilie et al. [105] used a BDD electrode for both tetracycline detection (by cyclic voltammetry) and degradation (by bulk electrolysis). Zhou et al. [106] focused on the pulse electrolytic oxidation of ciprofloxacin, showing high removal efficiency, while Song et al. [107] combined BDD electro-oxidation with ceramic ultrafiltration for simultaneous antibiotic degradation and membrane fouling control. Although these studies did not report detailed sensing performance (e.g., LOD, linear range), they illustrate the potential of BDD as a dual-function platform. Future research should aim to combine high-sensitivity detection with efficient degradation on a single BDD electrode.

Fifth, as monitoring demands shift from single-component to multi-component analysis, developing BDD-based multi-parameter sensing platforms is imperative. By integrating electrode arrays with different surface terminations (e.g., hydrogen-terminated, oxygen-terminated) or different functionalized modifications, combined with chemometric methods, simultaneous and accurate identification of heavy metals, phenolics, and emerging pollutants (e.g., antibiotics) can be achieved in a single measurement.

The design of such multi-parameter platforms must address several challenges: (i) the fabrication of arrays with reproducible electrode-to-electrode performance, (ii) the avoidance of cross-talk between adjacent sensing elements, and (iii) the development of chemometric algorithms that can deconvolute overlapping signals from multiple analytes. Principal component analysis (PCA) and partial least squares regression have shown promise in resolving overlapping voltammetric signals in other electrochemical sensor systems, and their application to BDD-based arrays represents a viable path forward [73]. Additionally, the inherent wide potential window of BDD makes it particularly suitable for multi-analyte detection, as the broad potential range allows multiple redox events to be captured without the constraints of a narrow solvent window [6,7,8].

Beyond the specific technical directions discussed above, three cross-cutting themes deserve particular attention for the practical translation of BDD sensors from laboratory prototypes to field-deployable devices [19,37]. First, electrode-to-electrode reproducibility must be addressed through optimized fabrication protocols and the development of quality control standards; the use of screen-printing and inkjet-printing methods for BDD electrode production offers a promising pathway to mass-producible, consistent devices [44,46]. Second, calibration stability under field conditions—where temperature fluctuations, ionic strength variations, and biofouling are inevitable—requires the development of self-calibrating protocols and protective surface coatings that maintain sensing performance over extended deployment periods [37]. Third, integration with portable electronics, including smartphone-based readout and wireless data transmission, will be essential for realizing the vision of decentralized, real-time water quality monitoring. Recent developments in miniaturized potentiostats and Bluetooth-enabled electrochemical sensors suggest that these technical barriers are surmountable, and BDD’s inherent durability makes it an ideal candidate for such applications [6,7,37].

In summary, the future of BDD-based electrochemical sensing lies in the rational integration of multi-scale structural design, data-driven optimization, real-world validation, and dual-function capabilities. The triple strategy of doping, modification, and annealing when systematically applied provides a solid foundation for achieving these goals.

## 7. Conclusions

This review has systematically examined the multi-scale structural regulation of boron-doped diamond (BDD) electrodes for electrochemical sensing of water pollutants, with a focus on overcoming the intrinsic “sensitivity-selectivity-stability triangle bottleneck”. We have proposed and evaluated a triple strategy, i.e., boron doping, nanomaterial modification (carbon and metal), and post-annealing treatment, as an integrated framework to break this bottleneck. Moderate boron doping (10^20^–10^21^ cm^−3^) enhances conductivity and sensitivity, while excessive doping narrows the potential window; carbon nanomaterials enlarge the electroactive area and lower detection limits but face stability challenges; metal nanoparticles introduce catalytic selectivity but are prone to aggregation; post-annealing treatment, while widely used as a preparative technique, has not been systematically studied in terms of its mechanism-parameter-performance relationships and synergistic coupling with doping and modification in BDD-based sensing applications. Annealing can lock the modified layer, reduce interfacial defects, release residual stress, and stabilize the electrode interface, though parameters must be carefully optimized to avoid graphitization or oxidation. The coupling follows a logical sequence: doping first (electronic basis), then modification (functionality), and finally annealing (stabilization).

Design guidelines for BDD-based electrochemical sensors:

Guideline 1: Target-oriented boron doping. Select the boron concentration according to the pollutant’s redox potential and the required sensitivity-selectivity balance. Low-to-moderate doping (10^19^–10^20^ cm^−3^) is generally suitable for phenolic compounds where a wide potential window is critical; moderate doping (10^20^–10^21^ cm^−3^) is recommended for heavy metal ions and antibiotics where enhanced conductivity is prioritized.
Guideline 2: Function-matched surface modification. Use carbon nanomaterials when the priority is to enlarge surface area and lower detection limit (e.g., trace heavy metals). Use metal nanoparticles when the priority is to improve selectivity through specific catalytic or affinity interactions (e.g., Bi for Pb^2+^/Cd^2+^, Au for Cr(VI)). Hybrid modification (carbon + metal) can achieve both high sensitivity and good selectivity.
Guideline 3: Annealing as a default stabilization step. Unless the modification layer is intrinsically stable (e.g., covalently bonded), post-annealing should be applied. Recommended starting conditions: 200–400 °C in inert or reducing atmosphere (Ar, H_2_) for 1–2 h. The annealing atmosphere should be chosen to preserve or tune the desired surface termination (H-terminated for high conductivity, O-terminated for hydrophilicity).
Guideline 4: Multi-parameter optimization. The three strategies are not independent. The optimal combination must be determined by considering the target pollutant, water matrix, and operational mode. Orthogonal experiments or AI-assisted screening can accelerate the identification of optimal combinations.
Guideline 5: Implementation sequence. Follow the sequence: optimize doping conditions first (setting the electronic basis of the electrode), then select and apply the appropriate modification layer (adding target-specific functionality), and finally apply annealing treatment (stabilizing the whole interface).

Current research gaps and future opportunities have been identified. Most notably, post-annealing has been rarely applied or systematically studied in BDD-based sensing. This represents a major opportunity for improving long-term stability without sacrificing sensitivity. In addition, the integration of sensing and degradation (dual-function BDD platforms) is an emerging concept that could transform water quality monitoring from passive detection to active remediation. Real-world validation, AI-assisted high-throughput screening, and multi-parameter sensing arrays are also promising directions. By moving from single-factor trial-and-error to multi-scale, multi-parameter synergistic design, BDD electrodes can achieve the high performance and long-term reliability required for practical water pollutant monitoring.

## Figures and Tables

**Table 1:** Performance priorities and recommended regulation strategies for different water pollutants.

Pollutant Type	Representative Examples	Pollutants Covered	Key Performance Needs	B Doping Level	Preferred Strategies	Recommended Annealing	Relevant Sections
Heavy metal ions	Pb^2+^, Cd^2+^, Zn^2+^, Cr(VI)	≥4 heavy metal species	Low LOD, high sensitivity, good selectivity	Moderate (10^20^–10^21^ cm^−3^)	Moderate B doping + metal nanoparticles (Bi, Au)	Strongly recommended	Section 3.1, Section 3.3 and Section 4
Phenolic compounds	Phenol, bisphenol A, acetaminophen, hydroquinone	≥4 phenolic species	Wide potential window, anti-fouling, repeatability	Low-to-moderate (10^19^–10^20^ cm^−3^)	Low-to-moderate B doping + carbon nanomaterials (graphene, CNTs)	Optional (case-dependent)	Section 3.1 and Section 3.2
Antibiotics and emerging contaminants	Tetracycline, sulfonamides, ciprofloxacin, PCB-77	≥4 emerging contaminant species	High selectivity, low LOD, long-term stability	Moderate (10^20^–10^21^ cm^−3^)	Moderate B doping + metal nanoparticles + aptamers/enzymes	Strongly recommended	Section 3.1, Section 3.3 and Section 4
Mixed pollutant systems	Heavy metals + organics	Multiple coexisting pollutants	Peak separation, anti-interference	Multi-scale composite	Multi-scale composite regulation	Case-dependent	Section 3.4 and Section 4.3

**Table 2:** Comparison of the mechanisms, advantages, and limitations of the three regulation strategies.

Strategy	Principle of Action	Main Advantages	Limitations	Representative References
Boron doping regulation	Modulates carrier concentration, potential window, and crystal structure	Improves conductivity and active site density; enhances sensitivity and selectivity	Increasing boron doping to enhance conductivity may narrow the potential window and reduce the relative density of active sites	[14,15,16,17,24]
Carbon nanomaterial modification	Increases effective area and builds conductive networks	Enlarges surface area; constructs conductive networks; improves sensitivity and lowers detection limit	Prone to aggregation and detachment; insufficient stability and reproducibility of the modification layer; easily fouled in complex water	[18,41,42,59,67,68]
Metal nanomaterial modification	Introduces catalytic active sites to enhance target reaction	Enhances catalytic activity; improves charge transfer; increases selectivity	Metal particles tend to aggregate, passivate, or dissolve; poor adhesion or aggregation over time reduces electrode lifetime	[17,20,76,78,79,84]
Combined regulation	Synergistic control of doping, carbon materials, and metal materials	Balances sensitivity, selectivity, and stability	Complex fabrication; interface mechanisms difficult to elucidate; high requirements for reproducibility	[28,83,85]

**Table 4:** Summary of BDD-based electrochemical sensors for organic pollutants (phenolic compounds, antibiotics, and emerging contaminants).

Electrodetype	B Doping Conditions	Modifier	Annealing Conditions	Detection Method	Target Analyte	Line Arrange	LOD	Sensitivity	Stability	Real Sample	Ref.
NG/BDD	High-temperature growth, B-rich conditions	In situ nanographite (growth-annealing)	In situ graphitization	DNPV	Acetaminophen	0.02–50 μM	5 nM	Not explicitly reported	~90% after 20 cycles	Commercial APAP tablets	Wang et al. 2021 [47]
NBDD	Bias-assisted HFCVD	None (nanostructured)	None	Not specified	Phenol	Not specified	Not specified	Higher than planar BDD	Stable after 50 cycles	Simulated wastewater	Shi et al. 2020 [9]
BDD (unmodified)	BDD film (B content ≈8000 ppm)	None	Not specified	DPV	Bisphenol A	0.44–5.2 μM	0.21 μM	1.48 ± 0.02 μA μM^−1^	Not reported	Tap water and lake water	Pereira et al. 2012 [48]
BDD + CTAB	BDD film (commercial BDD, 1000 ppm)	CTAB (surfactant)	Not specified	AdSV	BPA, hydroquinone	BPA 0.2–8.0 μg/mL; HQ 1.0–16.0 μg/mL	BPA: 0.05 μg/mL (2.19 × 10^−7^ M); HQ: 0.23 μg/mL (2.09 × 10^−6^ M)	BPA 3.269 μA mL μg^−1^; HQ 0.568 μA mL μg^−1^	Remains stable for 2 weeks.	Water samples	Hoshyar et al. 2021 [96]
Ni/BDD	BDD film, Ni-implanted	Ni (ion implantation)	850 °C, H_2_, 10 min	FIA with amperometry	Tetracycline	1.0–100 μM	10 nM	Calibration slope: 0.058 μA mM^−1^	Not reported	Not reported	Treetepvijit et al. 2005 [97]
Screen-printed BDD	Screen-printed diamond	None	425 °C, air	LSV	Ciprofloxacin	1–30 μM	0.588 μM	0.215 μA cm^−2^ μM^−1^	Not reported	Artificial urine diluted 100-fold	Matsunaga et al. 2020 [46]
AuNPs/BDD aptasensor	BDD film (details not specified)	Au nanoparticles + aptamer	Two-step sputtering/annealing	EIS	PCB-77	1.0 × 10^−15^–1.0 × 10^−11^ M	0.32 fM	Not reported	95% response retained after regeneration; stable over 10 days	lake water samples	Yuan et al. 2020 [28]

Note: BDD, boron-doped diamond; NG, nanographite; NBDD, nanostructured BDD; APAP, acetaminophen; BPA, bisphenol A; HQ, hydroquinone; CTAB, cetyltrimethylammonium bromide; DNPV/DPV/SW-AdSV/LSV/EIS/FIA, electrochemical detection methods; LOD, limit of detection.

**Table 5:** Summary of annealing treatment studies on BDD-based electrodes.

Electrode Type	Annealing Temperature	Annealing Time	Annealing Atmosphere	Structural Changes	Electrochemical Performance Effects	Ref.
BDD (boron-doped diamond films)	Systematic study (room temp to high temp)	60 min	Air	B_4_O-C oxy-boron-carbide formation; enhanced oxidation resistance	Improved thermal stability; hardness retention at high temperatures	Zhao et al. 2024 [26]
Cu-BDD	Temperature varied	15 min	N_2_	Improved Cu-BDD interface; reduced interfacial defects	Enhanced stability and performance for nitrate reduction	Kuang et al. 2021 [25]
Heavy B-doped BDD	600 °C	3, 10, 30, 90 min	Air	Surface etching; grain degradation with prolonged oxidation	SECM maps show local charge transfer heterogeneity increases with oxidation time	Ryl et al. 2020 [27]
NNBDD	800 °C	15 min	Air	Removal of non-diamond carbon phases; stabilized nanoneedle structure	Reduced background current; improved Pb^2+^ sensing with LOD of 0.32 μg/L	Yuan et al. 2023 [89]
G/SBDD	1000 °C	70 min	Vacuum	In situ graphene growth on BDD surface	Enhanced Pb^2+^ sensing; ~96% stability after 60 days in seawater	Pei et al. 2020 [41]
AuNPs/BDD	Two-step annealing	30 s × 2	Air	Stable AuNPs/BDD interface formation	Enabled aptasensor for PCB-77 with LOD of 0.32 fM	Yuan et al. 2020 [28]

## Data Availability

No new data were created or analyzed in this study. Data sharing is not applicable to this article.
